# Immunothrombosis versus thrombo-inflammation: platelets in cerebrovascular complications

**DOI:** 10.1016/j.rpth.2024.102344

**Published:** 2024-02-09

**Authors:** Lexiao Li, David Stegner

**Affiliations:** 1 Julius-Maximilians-Universität Würzburg, Rudolf Virchow Center for Integrative and Translational Bioimaging, Würzburg, Germany; 2 University Hospital Würzburg, Institute of Experimental Biomedicine, Würzburg, Germany

**Keywords:** blood platelets, cerebral venous thrombosis, immunothrombosis, ischemic stroke, thrombo-inflammation

## Abstract

A State-of-the Art lecture titled “Thrombo-Neuroinflammatory Disease” was presented at the International Society on Thrombosis and Haemostasis Congress in 2023. First, we would like to advocate for discrimination between immunothrombosis and thrombo-inflammation, as immunothrombosis describes an overshooting inflammatory reaction that results in detrimental thrombotic activity. In contrast, thrombo-inflammation describes the interplay of platelets and coagulation with the immunovascular system, resulting in the recruitment of immune cells and loss of barrier function (hence, hallmarks of inflammation). Both processes can be observed in the brain, with cerebral venous thrombosis being a prime example of immunothrombosis, while infarct progression in response to ischemic stroke is a paradigmatic example of thrombo-inflammation. Here, we review the pathomechanisms underlying cerebral venous thrombosis and ischemic stroke from a platelet-centric perspective and discuss translational implications. Finally, we summarize relevant new data on this topic presented during the 2023 International Society on Thrombosis and Haemostasis Congress.

## Essentials

• Ischemic stroke and cerebral venous thrombosis are both leading causes of disability.
• Both involve plasmatic coagulation, platelets, and immune cells, but pathomechanisms differ.
• Stroke is a thrombo-inflammatory disease with immune cell recruitment and barrier breakdown.
• Cerebral venous thrombosis is caused by thrombotic occlusion of cerebral veins and is a prime example of immunothrombosis.

## Graphical abstract

**Figure:**
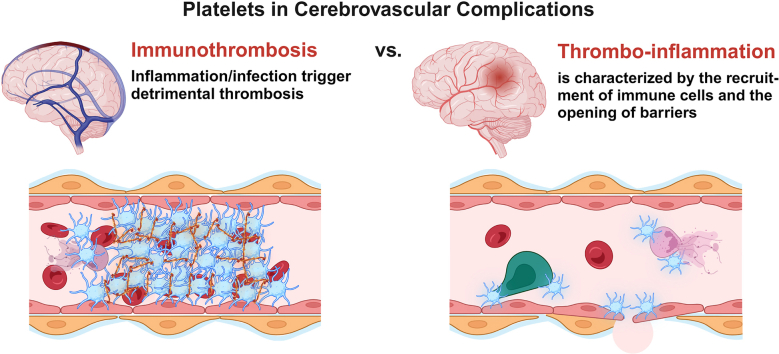

## 1 Introduction

Platelets, which derive from megakaryocytes within the bone marrow, are best known for their role in hemostasis and thrombosis. However, during the last 15 years, it has become clear that they also act as effector cells in inflammation, immune responses, development, and maintenance of barrier integrity [[1], [2], [3], [4], [5], [6], [7], [8], [9]]. While thrombosis and inflammation were long considered separate (patho-)physiological processes, overwhelming experimental and clinical evidence has demonstrated that they are indeed closely intertwined. These interactions can occur in 2 fundamentally different manners: immunothrombosis or thrombo-inflammation.

**Figure 1:**
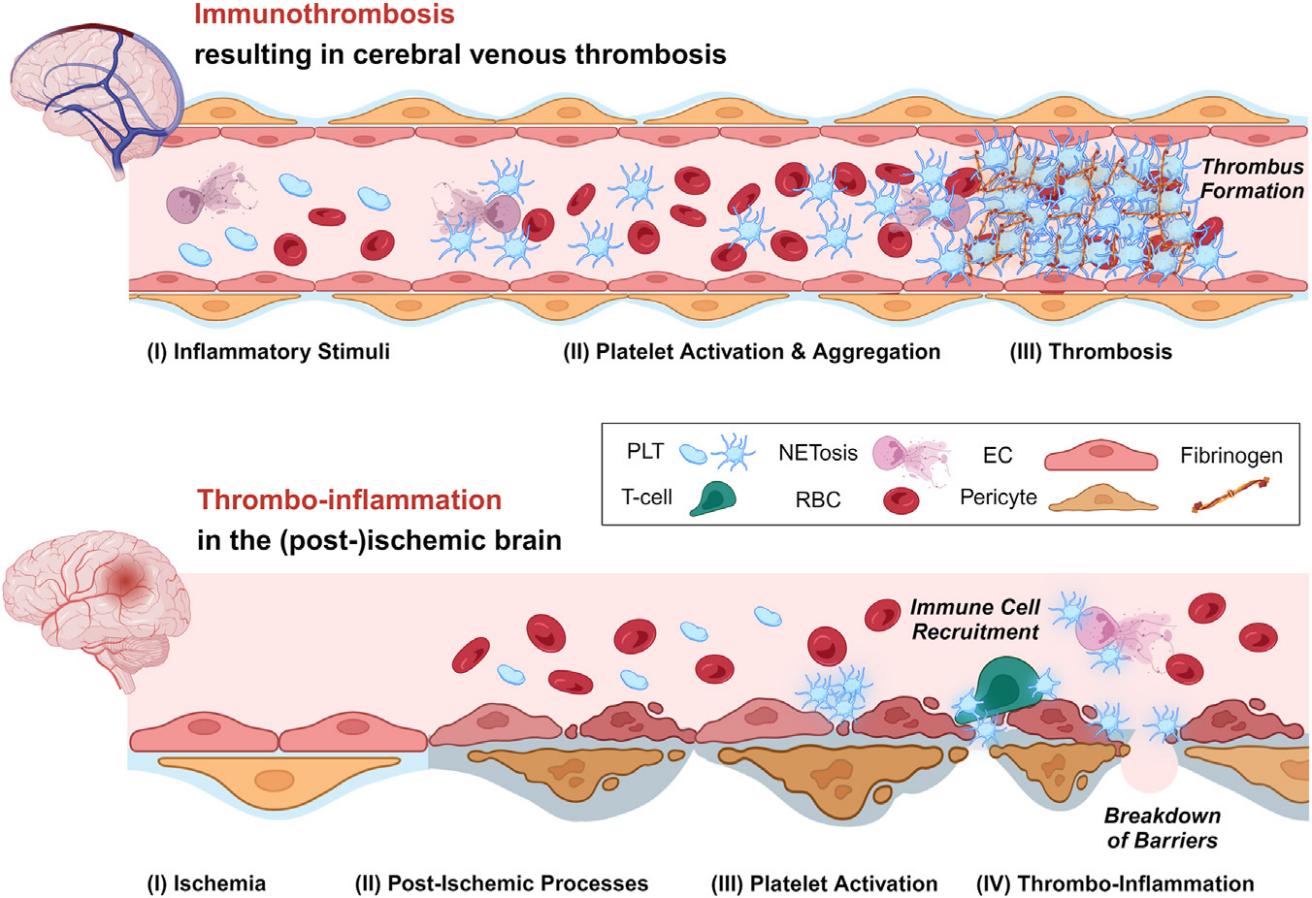
Essential pathophysiological differences underlying immunothrombosis and thrombo-inflammation in the cerebral vasculature. Both entities involve platelets, the plasmatic coagulation cascade, and immune cells. However, the main difference is the primary outcome: in the case of immunothrombosis, the primary outcome is the occlusion of a blood vessel due to platelet-involved thrombotic activity in response to initial inflammatory stimuli. One example of an immunothrombotic disease is cerebral venous thrombosis. In the context of thrombo-inflammation, the hallmarks are the recruitment of immune cells and the disturbance of barrier functions. This is best exemplified in the context of ischemic stroke, where non-classical functions of platelets are activated during post-ischemic processes. These platelet functions beyond thrombosis then contribute to thrombo-inflammation. EC, endothelial cell; NET, neutrophil extracellular trap; PLT, platelet; RBC, red blood cell.

The term immunothrombosis was introduced by Engelmann and Massberg [10] to describe immune-related thrombotic activity that prevents pathogen spreading. Meanwhile, the term immunothrombosis is used in a broader sense to describe infection or sterile inflammation-driven thrombotic events [11,12]. Importantly, the major pathological outcome is thrombosis, leading to insufficient supply of downstream tissue. Immunothrombosis should, however, not be confused with thrombo-inflammation, even though in both cases, an interplay between platelets and immune cells is observed (Figure 1). The term “thrombo-inflammation” was first proposed during studies of post-angioplasty restenosis due to platelet-leukocyte interactions [13]. Thrombo-inflammation was then recapitulated in studies involving platelet-eosinophil/neutrophil aggregation and platelet-promoted release of eosinophil/neutrophil oxidant products [14,15]. Subsequently, the Würzburg platelet group has used this term to describe the interplay of components of the thrombotic and inflammatory system driving ischemia/reperfusion (I/R) injury in acute ischemic stroke [1,[16], [17], [18], [19]]. In this process, initial platelet adhesion/activation pathways act in concert with key components of the contact pathway of plasmatic coagulation (factor [F]XII—kallikrein/kinin pathway) and immune cells to promote cerebral infarct growth [1]. Thus, the classical thromboembolic insult (brain artery occlusion) triggers a subsequent pathomechanism that again depends on platelets (and coagulation factors), which, however, in this context, use different signaling pathways and effector functions to orchestrate immune cell recruitment, edema formation, and eventually inflammatory tissue damage. Today, thrombo-inflammation is recognized as a major pathomechanism in a continuously growing number of arterial and venous disorders in different organs and disease states [2,20].

In the context of brain vasculature, typical immunothrombosis is seen in cerebral venous thrombosis (CVT), whereas thrombo-inflammation predominantly contributes to tissue damage/infarct progression following acute ischemic stroke. Thus, this review article will discuss more details about immunothrombosis and thrombo-inflammation based on these 2 prototypic diseases, respectively.

## 2 Cerebral Venous and Dural Sinus Thrombosis

Thrombosis takes place when an occlusive blood clot is developed in an artery or vein. Typical diseases caused by thrombosis include ischemic stroke, myocardial infarction, peripheral deep vein thrombosis, and its most dangerous complication, pulmonary embolism. The aftermath of thrombosis, in many cases, is fatal. Venous thrombosis harbors quite a few distinct features from arterial thrombosis, such as its growth at a low shear rate, frequent involvement of red blood cells, and a more prominent involvement of immune cells. Infections and inflammation are determined as risk factors for venous thrombosis, making the mechanisms of immunothrombosis a possible explanation for those “unprovoked” cases of venous thrombosis [21]. The emerging role of the hyperactivated immune system in venous thrombosis is represented by the involvement of neutrophil activation together with the formation and release of neutrophil extracellular traps (NETs) [22,23].

Cerebral venous and dural sinus thrombosis is a rare, atypical form of venous thrombosis that occurs either at a dural sinus (in particular, superior sagittal sinus), cortical vein, or the proximal jugular vein. Its occurrence in Western countries is estimated to be 4 per million in adults and 7 per million in neonates, with a mortality rate of 5% to 10% [24,25]. The risk factors of CVT can be genetic prothrombotic conditions, antiphospholipid syndrome, infections, head trauma, brain tumors, neurosurgery, anemia, and obesity, whereas the most significant risk factors are oral contraceptive use, pregnancy, and puerperium [26]. This indicates that hormonal changes contribute to the development of CVT and partly explains why women are more susceptible to CVT. CVT turns out to be the main cause of non-arterial stroke among young adults, accounting for 0.5% of all stroke cases. The symptoms of CVT can be highly heterogeneous, ranging from isolated headaches, visual/auditory disturbance, and hemiparesis to a comatose state, leaving the diagnosis of CVT a challenging task without the aid of medical imaging. Hydrocephalus, hemorrhage, intracranial hypertension, herniation, and seizure are common acute complications of CVT. Among several ongoing multicenter registries and clinical trials, the European guidelines on CVT diagnosis and management were updated in 2017 with recommendations made from an optimized level of evidence [27].

Conventional perspectives hold that the contribution of platelets appears to be more fundamental in arterial thrombosis versus venous thrombosis [28]. Thus, arterial thrombosis is deemed a platelet-associated disease, and antiplatelet drugs have been greatly recommended for the primary and secondary prevention of arterial thrombotic diseases. Platelets in the venous thrombi are found proportionally low, so venous thrombosis is commonly regarded as a coagulation cascade-related disease that should be treated with anticoagulants. However, mounting evidence suggests that platelets also play critical roles in the growth of venous thrombi. Systemic platelet activation has been determined in patients with acute venous thromboembolism [29]. The absence of platelets dampens the formation of thrombi in experimental venous thrombosis [30]. Using antiplatelet drugs such as aspirin and clopidogrel in murine venous stasis models or for patients subjected to orthopedic surgery, the risks of venous thrombosis and recurrence are consistently reduced [31,32]. The bleeding risk was not appreciable.

When it selectively comes to CVT, however, current knowledge about the underlying pathomechanisms is limited, which is in part due to the limitations of current animal models [33]. It appears that CVT is an immunothrombotic disease as thrombi from patients with CVT are rich in NETs [34], and platelet-driven NET formation is a crucial factor for the formation of venous thrombi in deep vein thrombosis [35,36]. Further evidence comes from the fact that the COVID-19 pandemic resulted in rising numbers of CVT cases [37,38], with immunothrombosis being one of the pathomechanisms underlying COVID-19 [11].

**Figure 2:**
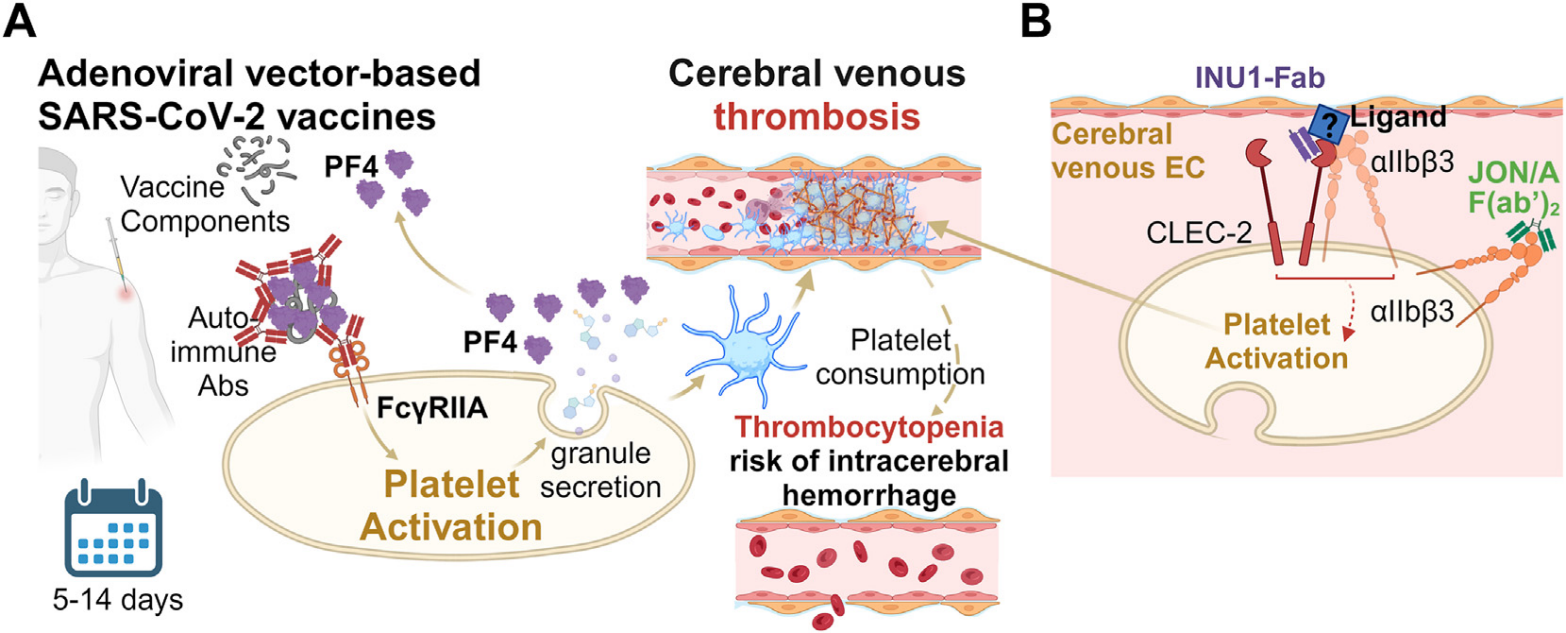
Cellular and molecular mechanisms regarding cerebral venous thrombosis (CVT) and vaccine-induced thrombotic thrombocytopenia (VITT). (A) One of the severe side effects of adenoviral vector-based SARS-CoV-2 vaccines is VITT. Briefly, the systemic inflammatory status due to vaccination can trigger a release of platelet factor 4 (PF4) from activated platelets. PF4 further forms a complex with vaccine components, which results in the engagement with anti-PF4 autoimmune antibodies (Abs). Once anti-PF4 antibodies are part of an immunocomplex, they are recognized by the FcγRIIA on the surface of platelets and neutrophils, leading to platelet activation and NETosis, ultimately resulting in thrombosis. As a severe complication of VITT, CVT occurs when the thrombi develop right in the brain venous system. The consumption of platelets further leads to secondary thrombocytopenia, which is particularly susceptible to intracerebral hemorrhage upon inflammatory conditions. (B) In an experimental CVT model, Fab-fragments of the anti-C-type lectin-like receptor-2 (CLEC-2) antibody INU1 apparently alter the conformation of CLEC-2. Consequently, CLEC-2 is able to interact with an unidentified ligand, which is enriched in cerebral veins. Then, cooperative signaling of CLEC-2 and α_IIb_β_3_ triggers platelet activation, resulting in a rapidly progressing CVT. Blocking CLEC-2 signaling or α_IIb_β_3_ (eg, using the anti-α_IIb_β_3_ JON/A-F[ab’]_2_-fragments) prevents CVT, indicating that this might be a novel therapeutic strategy. EC, endothelial cell.

In response to the COVID-19 pandemic, vaccines were rapidly developed, and unprecedented numbers of people were vaccinated within short periods of time. This allowed that even very rare side effects of the vaccines were detectable. A small fraction of individuals who received AstraZeneca/Oxford and Johnson & Johnson recombinant adenovirus vector-based SARS-CoV-2 vaccines developed a vaccine-induced immune thrombotic thrombocytopenia (VITT). This term was coined to highlight the similarities between VITT and heparin-induced thrombocytopenia (HIT) [39], as highly pathogenic anti–platelet factor 4 (anti-PF4) autoantibodies with heparin-independent platelet-activating properties are present in those patients (Figure 2A). As these antibodies trigger platelet activation via the FcγRIIA receptor, patients are usually treated with intravenous (i.v.) immunoglobulin to block FcγRIIA and a non-heparin anticoagulant [39]. The most severe consequence of VITT is CVT [40], which is frequently followed by intracranial hemorrhage. Moreover, the first clinical data indicates that anti-PF4 antibodies from patients with VITT binding to certain epitopes on PF4 are more likely to be associated with CVT than others [41]. However, it is not clear whether a second hit is required for VITT-caused thrombi to develop within the cerebral vasculature.

Recently, we revealed that the activation of C-type lectin-like receptor-2 (CLEC-2), whose signaling pathway is similar to that of FcγRIIA, can trigger CVT as well. Mice receiving a monovalent anti–CLEC-2 Fab-fragment (INU1) developed CVT within minutes after INU1-Fab i.v., which is accompanied by tonic-myoclonic seizures and thrombocytopenia [42]. Of note, thrombi were observed in various cerebral veins but not in other organs. The absence of CLEC-2 or inhibition of downstream signaling abolished all INU1-triggered effects, while heparin had only moderate effects. Interestingly, JON/A-F(ab’)_2_ fragments that block the fibrinogen binding site of α_IIb_β_3_ not only prevent thrombus formation but also prevent platelet activation, which argues for cooperative signaling of the 2 platelet receptors [42]. As INU1-Fab does not activate platelets *in vitro*, we speculate that it alters CLEC-2’s conformation, enabling the receptor to interact with a yet-to-be-identified ligand in cerebral veins, which upon binding to CLEC-2, and in concert with α_IIb_β_3_, causes CVT [42] (Figure 2B). Interestingly, we did not observe any signs of intracranial hemorrhage after α_IIb_β_3_ blockade, indicating that this might be a potential therapeutic strategy for the treatment of CVT.

## 3 Ischemic Stroke

Stroke remains one of the leading causes of disability and death worldwide. Eighty percent to 90% of stroke cases are ischemic, meaning that they are caused by a thrombus that occludes a large vessel, resulting in insufficient supply of the downstream tissue. Consequently, restoration of blood flow is the main therapeutic goal, which can be achieved by thrombectomy or i.v. thrombolysis (using recombinant tissue-type plasminogen activator). However, only a small proportion of patients are eligible to receive these therapies [43]. Moreover, even upon successful removal of the large vessel occlusion, only a fraction of patients benefit [44], underscoring the need for new therapeutic regimens and, hence, a better understanding of underlying pathomechanisms.

Animal models of ischemic stroke have been and still are instrumental in enhancing our understanding of infarct progression following ischemic stroke. Among those, the rodent model of transient middle cerebral artery occlusion (tMCAO), where typically an hour of brain focal ischemia is induced using a commercialized intraluminal monofilament, followed by a 24-hour reperfusion, is widely used [45]. Using this model on *Rag1*^−/−^ mice, which lack T and B cells, provided the first evidence that T cells contribute to infarct progression after ischemic stroke, as the adoptive transfer of T cells in *Rag1*^−/−^ mice fully restored infarct volumes [46]. A follow-up study confirmed these results and revealed that the effect of T cells in ischemic stroke is independent of specific T-cell receptors or costimulatory molecules [47]. Interestingly, studies using the permanent middle cerebral artery occlusion (MCAO) model, in which the filament is not removed, revealed that T cells already contribute to neuronal damage under occlusion [48], indicating that the detrimental activity of T cells is not limited to classical I/R injury. In line with these data, a multicenter trial in mice demonstrated smaller infarcts after cortical infarctions in mice that received an antibody blocking the central T-cell integrin α_4_β_1_ [49]. The T-cell effect, however, is apparently linked to platelets, as *Rag1*^−/−^ mice that receive T cells via an adoptive transfer after platelet depletion do not develop full-blown infarcts, indicating that platelets are orchestrating the detrimental effects of T cells [50]. One molecule linking the detrimental effects of platelets and T cells is the immune-receptor CD84, which is expressed on platelets and various immune cells, as mice lacking CD84 either on T cells or on platelets develop smaller infarcts following tMCAO [51].

Platelets have long been suspected of contributing to neuronal damage following ischemic stroke, as they accumulate at sites of cerebral infarctions [52] and are responsible for the initial insult (the occlusion of a large vessel). The common assumption has long been that platelets form aggregates within the microcirculation, preventing perfusion of the cerebral tissue. However, a seminal paper from Kleinschnitz et al. [16] has challenged this concept as it demonstrated that the blockade of α_IIb_β_3_, the central platelet integrin essential for aggregation, has no beneficial effect but, in contrast, results in intracerebral hemorrhages. The detrimental effects of α_IIb_β_3_ blockers in ischemic stroke were confirmed in clinical trials [53,54]. In line with these studies, we recently showed using light-sheet-fluorescence imaging that infarct progression precedes cerebral thrombosis [55], excluding thrombi as a central mechanism in ischemic stroke and underscoring that stroke is a thrombo-inflammatory disease (and not a thrombotic one). Remarkably, the difference in the efficacy of α_IIb_β_3_ blockade is one of the key points that illustrate the differences between immunothrombosis and thrombo-inflammation. Since cerebrovascular thrombo-inflammation is featured by the disturbance of the blood–brain barrier (BBB), we hypothesize that the activity of α_IIb_β_3_ is needed for the sealing of BBB breaches and the prevention of hemorrhagic transformation. In stark contrast, in the context of immunothrombotic CVT, blocking α_IIb_β_3_ was beneficial in an experimental model and not associated with bleeding complications [42].

**Figure 3:**
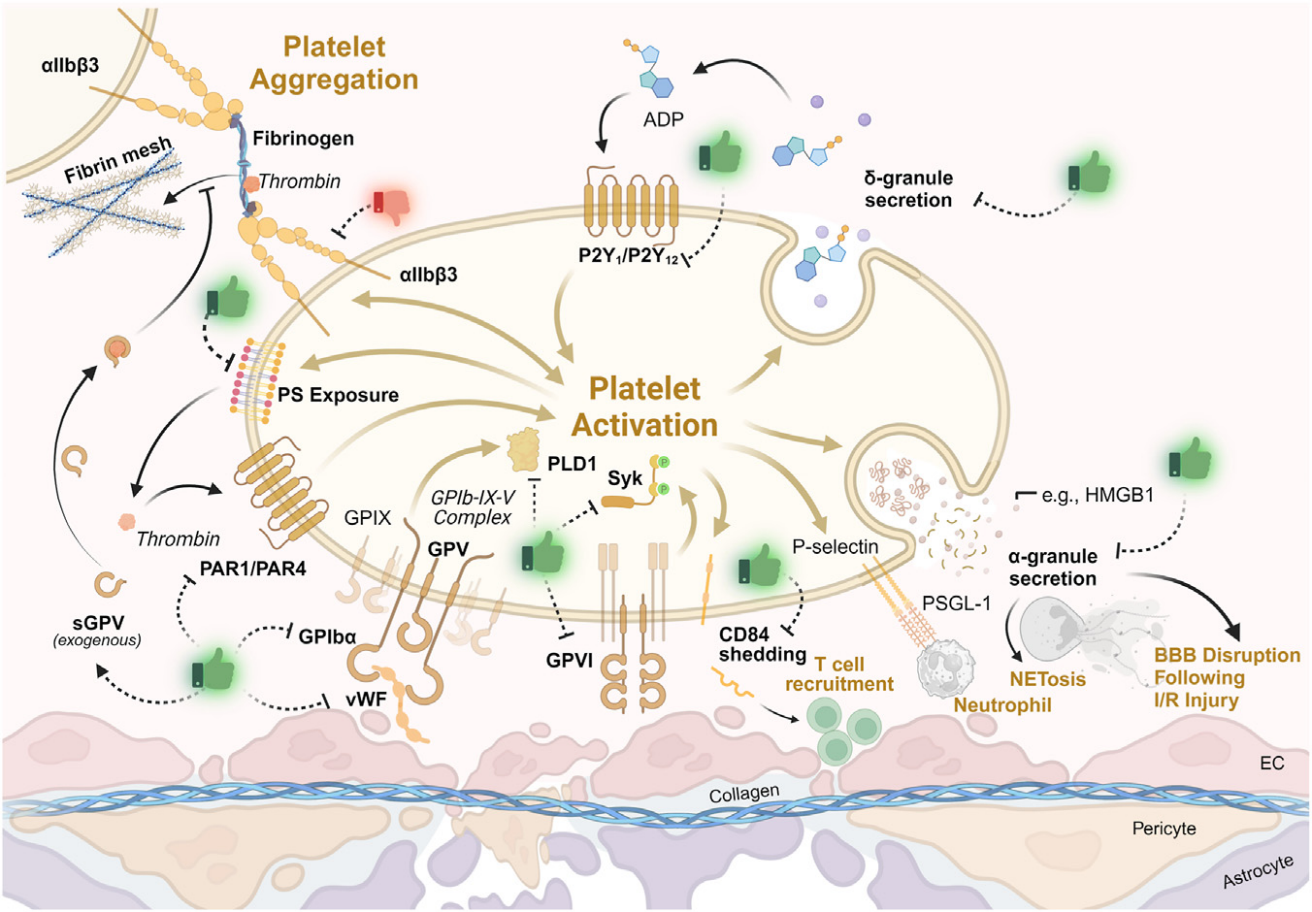
A simplified model of platelets as orchestrators of thrombo-inflammation (as seen in acute ischemic stroke). The (post-)ischemic endothelium exposes von Willebrand factor (VWF), allowing platelets to interact with VWF via glycoprotein (GP) Ibα, which is critical for infarct progression. Subsequently, platelets become activated via various signaling pathways, including GPVI, PARs, and ADP receptors, among others. Upon activation, platelets shed CD84, thereby recruiting T cells, expose P-selectin (a ligand for PSGL-1 on neutrophils) or phosphatidylserine (PS) (which promotes not only plasmatic coagulation but also supports NETosis), and activate their integrin receptors. On the one hand, plasmatic coagulation results in more thrombin generation, further activating platelets. On the other hand, thrombin cleaves GPV, and soluble GPV dampens fibrin generation. Activated platelets also secrete their granule content, resulting in the release of second-wave mediators such as ADP (from dense granules) as well as protein factors such as high-mobility group box 1 (HMGB1) (from α-granules) that contribute to the recruitment of neutrophils and trigger NETosis. Ultimately, this all converges in the recruitment of immune cells and the disruption of the blood–brain barrier (BBB), demonstrating that ischemic stroke is a thrombo-inflammatory disease. Corresponding strategies of potential intervention and aftermaths are indicated by thumbs up or down (depending on the outcome in experimental models) for future therapeutic developments. ADP, adenosine diphosphate; EC, endothelial cell; NETosis, NET activation and release; PAR, protease-activated receptor; PLD1, phospholipase D1; Syk, spleen tyrosine kinase.

Nevertheless, platelets are central contributors to infarct progression following ischemic stroke, as many platelet receptors and signaling pathways are involved in stroke development (Table 1 [16,^48^,^51^,^53^,^54^,[56], [57], [58], [59], [60], [61], [62], [63], [64], [65], [66], [67], [68], [69], [70], [71], [7]; Figure 3). As the final common pathway of platelet activation, integrin activation is of minor importance that raises the question: What platelet effector functions are of relevance instead (Table 2 [[76], [77], [78], [79], [80], [81], [82], [83]]; Figure 3)? Platelet degranulation is clearly a factor, as mice with ablated dense granule secretion (*Unc13d*^−/−^) develop smaller infarcts [77], potentially due to reduced platelet activation in the absence of adenosine diphosphate release. Likewise, *Nbeal2*^*-/-*^ mice, whose platelets lack α-granules, develop smaller infarcts [76], indicating that among the plethora of factors stored in these granules, some contribute to infarct progression. One critical component has been identified as high-mobility group box 1, which is elevated in patients with stroke, and mice lacking this protein in platelets develop smaller infarcts [84]. High-mobility group box 1 contributes to cerebral damage by promoting the formation of NETs [84]. Likewise, inhibition of NETs by neonatal NET-inhibitory factor [84] or DNase [85] reduces infarct volumes after tMCAO. Interestingly, platelets not only stimulate NET formation but also recruit neutrophils by exposing the neutrophil PSGL-1 ligand P-selectin and by exposing phosphatidylserine (PS) [86]. In a recent study [79], mice with cyclophilin D deficient platelets were investigated. These animals had less neutrophil infiltration and smaller infarct sizes following tMCAO (but not permanent MCAO), which was attributed to the abolished PS exposure in *CypD*^−/−^ platelets. Of note, however, the lack of the scramblase TMEM16F also abolished platelet PS exposure, but *Tmem16f*^*-/-*^ mice displayed unaltered infarct volumes following tMCAO [80]. On the other hand, blocking PS by annexin A1 [81] or lactadherin [82] reduced infarct volumes in experimental stroke models, supporting the role of PS in infarct progression. Besides secretion or PS exposure, receptor shedding is another mode of platelets to modulate inflammation, as CD84 shedding fosters T-cell recruitment into the ischemic brain [51]. A simplified model of platelets as orchestrators of thrombo-inflammation is depicted in Figure 3.

**Table 1:** Overview of platelet receptors involved in the pathogenesis of ischemic stroke.

Receptor/ligand	Model	Intervention	Outcome	Reference
α_IIb_β_3_	tMCAO (M)	Blockade	Unaltered infarct sizes, intracerebral hemorrhages	[16]
α_IIb_β_3_	H	Blockade	No benefits of treatment, higher rate of intracranial hemorrhages	[53,54]
β1-integrin	tMCAO (M/R)	Blockade	Bigger infarct volumes and worsened outcome with less angiogenesis and vascular remodeling	[56,57]
CD84	tMCAO (M)	Genetic deficiency	Smaller infarct sizes, better neurologic outcome with less immunocyte extravasation	[51]
GPIbα	tMCAO (M/R)	Blockade	Smaller infarct sizes, reduced apoptosis, reduced neuroinflammation, better neurologic outcome without inducing intracerebral hemorrhage	[16,58]
GPIbα	tMCAO (M)	Genetic deficiency	Smaller infarct sizes, better neurologic outcome in the absence of the GPIbα ectodomain or in case of defective signaling (phospholipase D1 ablation)	[59,60]
GPIbα	pMCAO (M)	Blockade	Smaller infarct sizes, reduced apoptosis, less immunocyte extravasation	[48]
GPV	tMCAO (M)	Recombinant protein	Smaller infarct sizes, better neurologic outcome	[61]
GPVI	tMCAO (M)	Receptor depletion	Smaller infarct sizes, better neurologic outcome—similar results upon blockade/deficiency of the downstream kinase Syk	[16,62]
GPVI	tMCAO (M)	Recombinant protein (competitive inhibition)	Smaller infarct sizes, better neurologic outcome without inducing intracerebral bleeding	[63]
GPVI	pMCAO (M)	Receptor depletion	Smaller infarct sizes with reduced immunocyte extravasation	[64]
PAR1	pMCAO (M) tMCAO (R)	Genetic deficiency RNA silencing	Smaller infarct sizes associated with decreased plasmin and thrombin activities, better functional outcome	[65,66]
PAR4	tMCAO (M)	Genetic deficiency	Smaller infarct sizes, better neurologic outcome with less platelet/endothelial and leukocyte/endothelial interactions and less BBB leakage	[67]
PAR4	tMCAO (M)	Blockade	Smaller infarct sizes, outcome negatively associated with the platelet-neutrophil aggregate formation	[68]
P2Y_1_	tMCAO (M/R)	Genetic deficiency Blockade	Improvement in post-ischemia cognitive impairment with diminished neuroinflammation	[69]
P2Y_12_	tMCAO (M)	Blockade	Smaller infarct sizes, better neurologic outcome with a better cerebral blood flow	[70]
P2Y_12_	H	Blockade	Safe and effective for acute stenting upon acute ischemic stroke	[71]
VWF	Guinea pig	Blockade	Smaller infarct sizes with better cerebral blood flow, void of intracerebral hemorrhage	[72]
VWF	tMCAO (M)	Genetic deficiency	Smaller infarct sizes, better neurologic outcome	[73,74]
VWF	tMCAO (M)	Smaller multimers due to rhADAMTS-13	Smaller infarct sizes, better neurologic outcome without inducing intracranial hemorrhage conversion	[75]

BBB, blood–brain barrier; GP, glycoprotein; H, human/clinical study; M, mouse; pMCAO, permanent middle cerebral occlusion; R, rat; Syk, spleen tyrosine kinase; tMCAO, transient middle cerebral artery occlusion; VWF, von Willebrand factor.

**Table 2:** Overview of platelet effector functions involved in the pathogenesis of ischemic stroke.

Effector function	Model	Intervention	Outcome	Reference
α-granule secretion	tMCAO (M)	*Nbeal2*^−/−^	Smaller infarct sizes, better neurologic outcome	[76]
δ-granule secretion	tMCAO (M)	*Unc13d*^−/−^	Smaller infarct sizes, better neurologic outcome without signs of ICH	[77]
Granule secretion	tMCAO (M)	*Nbeal2*^−/−^/*Unc13d*^-/-^	Smaller infarct sizes, however, more frequent ICH resulting in higher mortality	[78]
PS exposure	tMCAO (M)	*CypD*^−/−^	Smaller infarct sizes, better neurologic outcome with reduced platelet-neutrophil aggregates and neutrophil recruitment	[79]
PS exposure	tMCAO (M)	*Tmem16f*^−/−^	Unaltered infarct volumes and neurologic outcome	[80]
PS exposure	tMCAO (M)	Blockade with annexin A1	Smaller infarct sizes, better neurologic outcome with less platelet activation/aggregation and less thrombosis	[81]
PS exposure	dMCAO (M)	Blockade with lactadherin	Smaller infarct sizes, better neurologic outcome with preserved vascular and BBB integrity, less neurodegeneration, and less neuroinflammation	[82]
Serotonin	tMCAO (M)	*5Htt*^−/−^	Reduced platelet serotonin content did not affect stroke outcomes	[83]

BBB, blood–brain barrier; dMCAO, distal middle cerebral occlusion; ICH, intracranial hemorrhage; M, mouse; PS, phosphatidylserine; tMCAO, transient middle cerebral artery occlusion.

## 4 International Society on Thrombosis and Haemostasis 2023 Congress Report

Among advances in fundamental, translational, population, and clinical studies relevant to society, the International Society on Thrombosis and Haemostasis (ISTH) has highlighted the most recent advances regarding CVT- or VITT-relevant immunothrombosis during its 2023 Congress. In addition, several presentations provided new insights into pathomechanisms underlying ischemic stroke, which is a paradigmatic thrombo-inflammatory disease. Here, we highlight selected presentations of the ISTH 2023 Congress regarding cerebral immunothrombosis and thrombo-neuroinflammation.

### 4.1 ISTH 2023—advances regarding CVT and VITT

CVT represents a considerable fraction of venous thrombotic events in pediatric patients, which have been associated with poor outcomes. Manco-Johnson et al. [87] assessed the current outcomes of CVT in pediatric thrombosis centers. They found that mortality and thromboembolism recurrence have become scarce in pediatric cases of CVT. By contrast, long-term neurologic deficits prevail in these cohorts, including epilepsy, motor disorders, speech, and learning difficulties [87].

As elucidated above, VITT is a very rare but life-threatening adverse complication of adenoviral vector-related vaccines against the SARS-CoV-2 virus, characterized by thrombocytopenia and, in some cases, CVT. Anti-PF4 antibody immunocomplexes are the main trigger of platelet activation in not only VITT but also classical HIT. Wang et al. [88] have generated recombinant anti-PF4 antibodies derived from the sera of patients with VITT and presented their initial characterization at the congress. The recombinant human VITT anti-PF4 immunoglobulin G (IgG) will serve as a pivotal research tool for understanding the pathophysiology of VITT.

The laboratory-based differential diagnosis between VITT and HIT appears to be a challenging task. HIT anti-PF4 antibodies generally last 50 to 80 days after heparin exposure, while VITT anti-PF4 antibodies turn out to be detectable for longer periods of time [89]. Rollin et al. [90] established a competitive enzyme immunoassay with a monoclonal anti-PF4 F(ab’)_2_ fragment to discriminate between HIT and VITT antibodies in patient samples. Along the same lines, Schönborn et al. [91,92] have developed a set of novel assay prototypes to distinguish the anti-PF4 IgG of patients with VITT from those of patients with HIT. The identification of VITT-specific anti-PF4 IgG further benefits the prospective diagnosis of an overlooked cohort developing anti-PF4 IgG and severe immunothrombosis without the preceding exposure to heparin or vaccination because the type of anti-PF4 IgG in these patients turns out to be predominantly VITT-specific. Moreover, Nazy et al. [93] correlated the susceptibility of cerebral venous sinus thrombosis among patients with VITT with a special subset of anti-PF4 IgG, which binds to specific epitopes on the PF4 tetramer, enabling them to trigger platelet activation in the absence of exogenous PF4.

Nocella et al. [94,95] performed a series of studies using direct samples from patients with VITT. They determined that the appreciable thrombosis in these patients is preceded by PAD4-mediated NET formation and massive leukocyte-platelet interactions [94,95], which underscores that VITT and CVT are immunothrombotic diseases. In accordance with these findings, Wang et al. [96] assessed inflammatory and thrombotic parameters in patients with VITT and observed a correlation between disease severity and hyperinflammation, NETosis, and extensive cell death resulting in lymphocyte loss. Martins-Gonçalves et al. [97] suggested a positive association of NLRP3-driven inflammasome activation in patients with VITT, indicating an inflammatory process that underlies this disorder of immunothrombosis. Lombardi et al. [98] and Pallucci et al. [99] compared the effect of adenovirus-based and messenger RNA–based SARS-CoV-2 vaccines on platelet immune cell interactions and observed more sustained interactions upon messenger RNA–based vaccination, while adenovirus-based vaccines triggered antiviral-like responses resulting in platelet clearance. The authors speculate that more sustained platelet-B cell interactions might result in more efficient vaccination responses. Meanwhile, Keragala et al. [100] performed a pioneering investigation on the fibrinolytic system in patients with VITT. They found that VITT is associated with a remarkable hyperfibrinolytic state in addition to its well-established characteristic thrombosis, thrombocytopenia, and the generation of anti-PF4 IgG, which they attribute to a yet-to-be-identified plasma factor capable of reinforcing tissue-type plasminogen activator–associated plasminogen activation [100].

It has been repeatedly reported by a host of studies that anti-PF4 antibodies are also detectable and tangibly elevated in the plasma among non-VITT cohorts vaccinated by an adenovirus-based vaccine (represented by Guerra et al. [101]). The clinical significance of non-pathogenic anti-PF4 IgG remains obscure. It is speculated that anti-PF4 antibodies are not the only culprit for VITT. This hypothesis is supported by another study presented during the ISTH Congress. Rauova et al. [102] have developed an experimental mouse model based on vaccination with adenovirus-based vaccines to recapitulate the major clinical and pathologic features of VITT. Notably, mice developing VITT-like symptoms had only slightly elevated levels of anti-PF4 antibodies, while a more pronounced increase of circulating antibodies directed against the platelet-derived chemokine NAP2 was observed. The authors speculated that anti-NAP2 antibodies might contribute to the development of VITT.

Zlamal et al. [103] assessed inhibitors of the spleen tyrosine kinase (Syk), which mediates tyrosine phosphorylation downstream of (hem)ITAM receptors like FcγRIIa, with regard to their effect on VITT antibody-mediated platelet activation. In fact, 2 of the 3 tested Syk inhibitors dampened platelet activation [103]. Consequently, the authors suggest Syk inhibition as a potential therapeutic approach, which is in line with studies using a mouse model of CVT [42].

### 4.2 ISTH 2023—advances regarding pathomechanisms underlying ischemic stroke

In an experimental study, the effects of ambient light and red light exposure were investigated. The authors reported that red light exposure correlates with reduced platelet aggregation, activation, and particularly reduced platelet-dependent NET formation, culminating in a reduction of post-ischemia brain tissue damage [104]. However, we would kindly remind our readers that many of the studies mentioned in the ISTH Congress Report have not been peer-reviewed. In thrombo-inflammatory disease settings, platelets orchestrate immune cell recruitment (eg, neutrophil recruitment) and regulate vascular functions. Mechanistically, new processes of how platelets can regulate thrombo-inflammation were presented during the ISTH 2023 Congress. A new platelet-derived membrane structure called platelet-derived integrin and tetraspanin-enriched tethers (PITTs) was presented [105], which was detected on lung endothelium following pulmonary inflammation and neutrophils interacted with PITTs, indicating a potential role in immune cell recruitment. Of note, fewer PITTs were observed in von Willebrand factor (VWF)–deficient animals. Albeit the authors did not assess the role of PITTs in experimental stroke, it is tempting to speculate that PITTs might be involved in VWF-dependent immune cell recruitment following ischemic stroke, as post-ischemic endothelial cells expose VWF[106], and previous studies have shown that platelet α_IIb_β_3_ can modulate neutrophil activities [107,108].

Another mechanism for how platelets recruit neutrophils could be the secretion of the lipid mediator leukotriene B_4_ (LTB4, [109]). Hackenbroch et al. [109] presented data from mice lacking the hydrolase Lta4h that is responsible for the last step of LTB4 biosynthesis and reported no alteration in hemostasis or classical platelet assays but reduced neutrophil recruitment in inflammatory disease models with mice lacking platelet-derived LTB4.

The interplay of platelets and neutrophils in the context of ischemic stroke is a focus of Denorme’s research [68,84], who presented his latest study at the ISTH Congress [68,110]. Previous studies revealed that a dimorphism within the major thrombin receptor, protease-activated receptor 4 (PAR4), on platelets accounts for differences in platelet reactivity between races as Black people carry more frequently the more reactive variant PAR4^Thr120^, while in White individuals, the PAR4^Ala120^ variant is dominant [111]. In line with the elevated platelet reactivity of the Thr120-variant, this variant was associated with a higher incidence of ischemic stroke and a worsened outcome [68,110]. To explore the underlying mechanisms, the authors capitalized on humanized mice expressing either hPAR4^Thr120^ or hPAR4^Ala120^. The hPAR4^Thr120^ mice show worsened post-stroke outcomes, where significant thrombo-inflammation metrics are engaged, including platelet-neutrophil interactions and NETs. Notably, these post-stroke phenotypes seen in hPAR4^Thr120^ mice are unresponsive to the mainstay antiplatelet drugs such as ticagrelor (P2Y_12_ antagonist) and aspirin (TxA_2_ synthesis inhibitor). Instead, post-stroke outcomes in hPAR4^Thr120^ mice are considerably improved following the therapeutic strategy of blocking platelet-neutrophil interactions with an anti-P-selection antibody. These novel experimental findings highlight the fundamental role of thrombo-inflammation in the pathophysiology of cerebral I/R injuries and point toward novel therapeutic avenues.

As elucidated above, we favor a model in which the early events of hemostasis (ie, platelet adhesion and activation) are key players mediating proinflammatory signaling pathways following ischemic stroke. In contrast, classical platelet aggregation is not involved in these thrombo-inflammatory processes but may instead help prevent inflammatory hemorrhagic conversion. In line with this concept, Janus-Bell et al. [112] presented a study using platelet-specific deficiency (using the PF4-Cre system) of either β1- and/or β3-integrins. Interestingly, only mice whose platelets lacked all integrins displayed brain hemorrhages 6 hours after transient MCAO, indicating that the 2 β1- and β3-integrins can, in part, compensate for one another in maintaining vascular integrity following experimental stroke.

Once the BBB is disturbed, platelets could leave the vasculature, reach the parenchyma, and interact with cells of the neurovascular interface they would usually not encounter. Bourne et al. [113] reported that platelets can be found interacting with microglia following photothrombosis-induced stroke. As platelets modulate the inflammatory profile of microglia *in vitro*, the authors speculate that these interactions modulate post-stroke neuroinflammation, albeit it remains to be determined how frequently these interactions occur.

## 5 Future Directions

The last decade has revealed that platelets orchestrate far more than hemostasis and thrombosis; they are versatile effectors cells in a wide variety of (patho-)physiological processes. While thrombosis and inflammation were long considered 2 separate (patho-)physiological processes, the concept of thrombo-inflammation recognizes that they are not only closely intertwined but often also interdependent. Platelets have a key role in this concept as central orchestrators of immune cell trafficking and activation, vascular barrier function, and organ integrity. Correct concepts of disease progression and knowledge of the detrimental factors will be essential, as it appears likely that molecules critically involved in thrombo-inflammation may be less relevant in immunothrombosis and vice versa. The advent of advanced imaging techniques and the prevalence of high-throughput data science will certainly uncover further roles of platelets and deepen our understanding of the underlying mechanisms providing new targets for therapeutic interventions.
